# Multi‐Layer Transcriptomic Comparison Across Human, Pig, and Mouse Informs Tissue‐ and Trait‐Specific Animal Model Selection

**DOI:** 10.1002/advs.77881

**Published:** 2026-09-27

**Authors:** Jingwen Dou, Yong Liao, Xiong Shen, Xin Huang, Zhenshuang Tang, Yuwei Gou, Huajun Zhou, Jingya Xu, Hong Liu, Yue Wang, Keke Liang, Jia Liu, Xiao Zhang, Chenyao Li, Shangjian Wang, Liangliang Fu, Lilin Yin, Xinyun Li, Shuhong Zhao, Xiaolei Liu, Jingjin Li, Yuhua Fu

**Affiliations:** ^1^ Key Laboratory of Agricultural Animal Genetics Breeding and Reproduction Ministry of Education Huazhong Agricultural University Wuhan Hubei China; ^2^ Yazhouwan National Laboratory Sanya China; ^3^ Frontiers Science Center for Animal Breeding and Sustainable Production Ministry of Education Wuhan China; ^4^ Hubei Hongshan Laboratory Wuhan Hubei China; ^5^ Hubei Livestock Breeding Technology Innovation Center Wuhan China; ^6^ National Engineering and Technology Research Center for Livestock Wuhan China

**Keywords:** animal model selection, cross‐species transcriptomics, gene co‐expression conservation, human complex traits

## Abstract

Animal models are fundamental to biomedical research, yet the relative suitability of pigs and mice remains unclear across tissues, regulatory layers, and phenotype domains. Here, we assembled a large‐scale, tissue‐matched transcriptomic resource comprising more than 16 000 RNA‐seq samples from 15 corresponding human, pig, and mouse tissues, and established a unified framework integrating global co‐expression conservation, co‐expression network architecture, tissue‐specific programs, 3D genome architecture, and human complex‐trait genetics. Using 14 910 one‐to‐one orthologous genes, we found that human‐pig pairs showed higher global co‐expression similarity than human‐mouse pairs in most tissues, with exceptions including skin, pancreas, and kidney. Integration with 176 human complex traits further revealed distinct phenotypic associations of species‐preferred patterns: human‐pig‐preferred genes showed preferential associations with cardiometabolic, biochemical, and blood‐related traits, whereas both‐conserved genes showed stronger associations with neuropsychiatric, cognitive, and behavioral phenotypes. Together, our results demonstrate that cross‐species similarity is hierarchical and context‐dependent, varying across tissues, regulatory layers, and trait domains. Rather than supporting a universally optimal model organism, our study provides a comparative framework for prioritizing animal models based on regulatory conservation, together with an interactive portal for gene‐centered exploration across humans, pigs, and mice.

## Graphical abstract

We integrated RNA‐seq data from humans, pigs, and mice across 15 matched tissues based on 14 910 one‐to‐one orthologous genes. Cross‐species similarity was systematically evaluated across multiple regulatory layers, including global co‐expression conservation, co‐expression networks, tissue‐specific programs, and 3D genome architecture. These analyses were further integrated with human complex‐trait genetics and implemented as an interactive web resource for gene‐centered exploration.

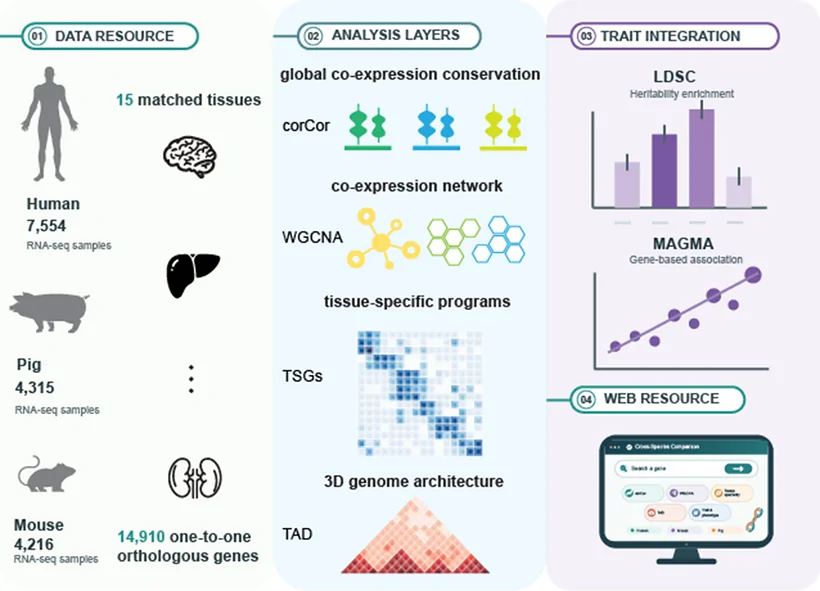

## 1 Introduction

Animal models have been indispensable for advancing our understanding of biology, physiology, and medicine. Among them, mice have long served as the primary model for human disease and drug development, owing to their genetic tractability, short generation time, and shared biological pathways with humans [1, 2, 3]. However, mice also exhibit notable anatomical and physiological differences from humans and frequently show distinct responses to drugs due to differences in metabolism, pharmacokinetics, and pharmacodynamics [4, 5, 6, 7]. In contrast, pigs share many anatomical, physiological, and gut microbiome features with humans [8]. Consequently, pigs are increasingly recognized as valuable complements to mice and as alternative models for specific biomedical applications, particularly in studies of human organ development, transplantation, surgical innovation, and therapeutic evaluation [9, 10, 11]. In parallel, major initiatives such as pigGTEx [12, 13], IAnimal [14], and related projects [15, 16, 17] have generated extensive transcriptomic and regulatory resources in pigs, creating an unprecedented opportunity for systematic cross‐species transcriptomic comparisons.

Cross‐species transcriptomic analyses have provided important insights into conservation and divergence of gene regulation across mammals, revealing evolutionary constraints, tissue‐specific functions, and lineage‐specific regulatory divergence [18, 19, 20, 21]. Previous studies have compared human and pig transcriptomes to investigate expression divergence and disease relevance [22, 23, 24, 25], while others have focused on human‐mouse comparisons to evaluate the translational utility of mouse models [26, 27]. More recently, several studies have extended these analyses beyond gene expression levels to co‐expression networks and regulatory programs, suggesting that pigs may more closely resemble humans than mice in specific tissues or regulatory contexts, such as the brain [28]. However, most existing studies remain restricted to individual tissues, limited sample collections, or a single regulatory dimension. As a result, current comparisons often provide incomplete or dimension‐specific views of cross‐species similarity and offer limited guidance for determining when pigs or mice may better represent human biology. Moreover, the degree of similarity may vary substantially across tissues and biological processes. Therefore, a unified framework that simultaneously evaluates these distinct dimensions across humans, pigs, and mice is needed to move beyond global species‐level comparisons and toward a more context‐aware understanding of model suitability.

**FIGURE 1:**
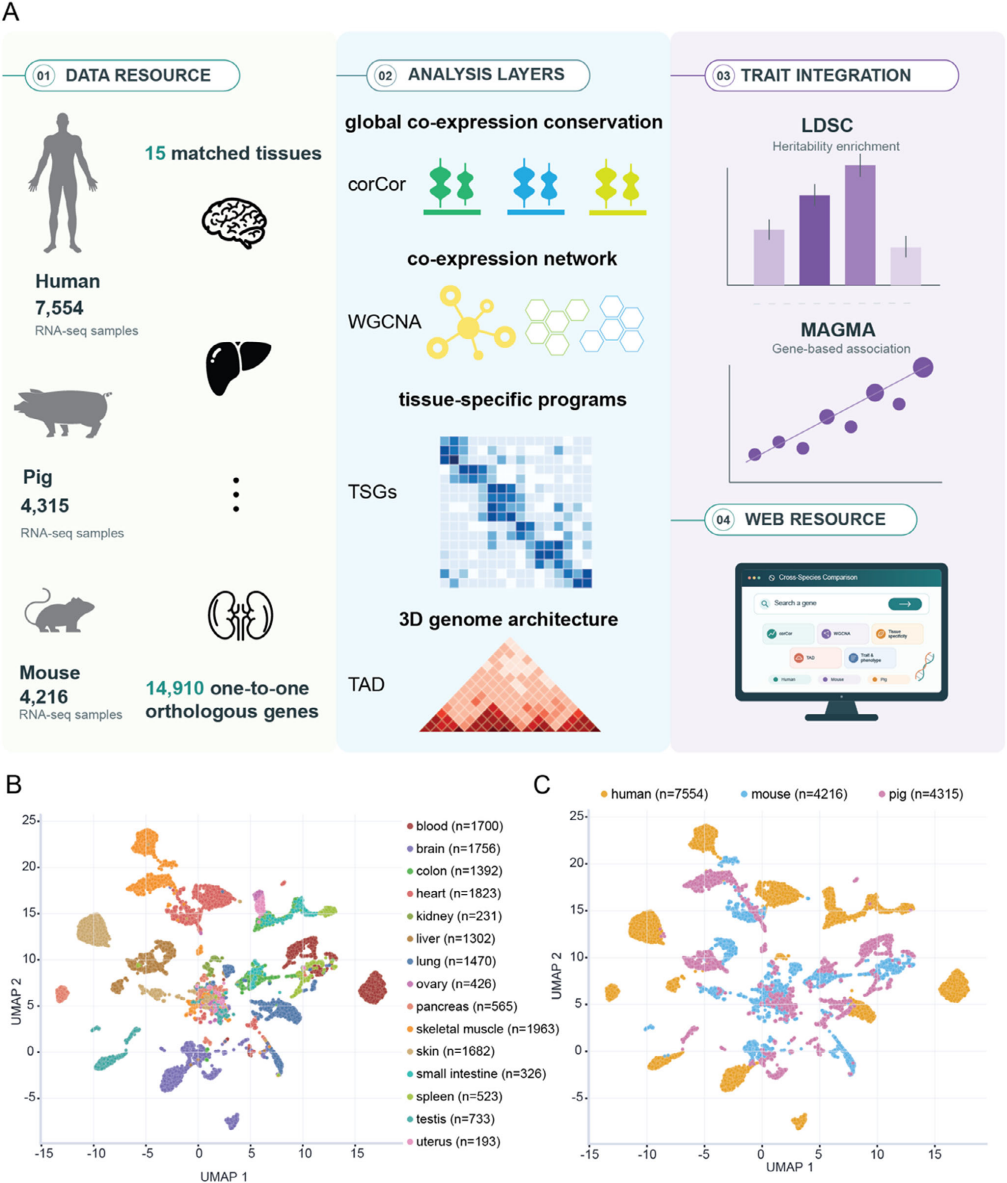
Study design and global transcriptomic landscape across humans, pigs, and mice. (A) Schematic overview of the study design. A large‐scale transcriptomic compendium comprising > 16 000 RNA‐seq samples across 15 matched tissues was assembled. Cross‐species similarities and divergences were systematically evaluated across multiple regulatory layers, including global co‐expression conservation measured by the correlation of the correlation coefficient (corCor); module preservation based on weighted gene co‐expression network analysis (WGCNA); tissue‐specific gene programs (TSGs); and topologically associating domains (TADs), followed by integration with human complex‐trait genetics. (B) UMAP visualization of 16 085 transcriptomic samples from humans, pigs, and mice based on 14 910 one‐to‐one orthologous protein‐coding genes. (C) The same UMAP embedding as in (B), colored by species.

To address this gap, we assembled a large‐scale, tissue‐matched transcriptomic resource encompassing more than 16,000 RNA‐seq samples across 15 corresponding tissues from humans, pigs, and mice. Using this resource, we establish a comparative framework integrating global co‐expression similarity, co‐expression network architecture, tissue‐specific gene programs, higher‐order chromatin organization, and associations with human complex traits (Figure 1A). Rather than assuming that a single species is universally more similar to humans, we systematically evaluate how transcriptomic conservation varies across tissues, regulatory layers, and human complex traits. Our analyses reveal that cross‐species similarity is highly context‐dependent and provide a multi‐layer resource for comparative model evaluation in functional transcriptome, comparative biology, and translational research.

## 2 Results

### 2.1 Assembly of a Large‐Scale, Tissue‐Matched Transcriptomic Resource Across Humans, Pigs, and Mice

To establish systematic cross‐species comparisons of transcriptional profiles, we assembled a large‐scale tissue‐matched transcriptomic resource comprising more than 16 000 RNA‐seq samples from humans, pigs, and mice (7554, 4315, and 4216 samples, respectively; Table S1). This resource spans 15 anatomically matched tissues representing major organ systems, including the nervous, circulatory, digestive, reproductive, and immune systems. To ensure direct comparability, we focused on 14 910 one‐to‐one orthologous protein‐coding genes shared among humans, pigs, and mice (Table S2). These genes represent approximately 75%, 67%, and 68% of annotated protein‐coding genes in humans, pigs, and mice, respectively. This matched orthologous gene set provided a common gene set for evaluating conservation and divergence of transcriptional regulation across species.

Based on the expression profiles of the above 14 910 one‐to‐one orthologous genes, we assessed their global transcriptomic relationships, the resulting UMAP showed that samples were organized primarily by tissue identity rather than by species (Figure 1B,C). For example, anatomically or functionally related tissues, including heart and skeletal muscle, colon and small intestine, and ovary and uterus, were positioned in neighboring regions of the UMAP space. Although the extent of cross‐species overlap varied among tissues, this tissue‐dominant organization is consistent with broad conservation of major tissue‐associated transcriptional programs among humans, pigs, and mice. These observations provided a rationale for subsequent tissue‐matched analyses of cross‐species similarity across multiple regulatory layers.

### 2.2 Global Gene Co‐Expression is More Conserved Between Humans and Pigs Than Between Humans and Mice

**FIGURE 2:**
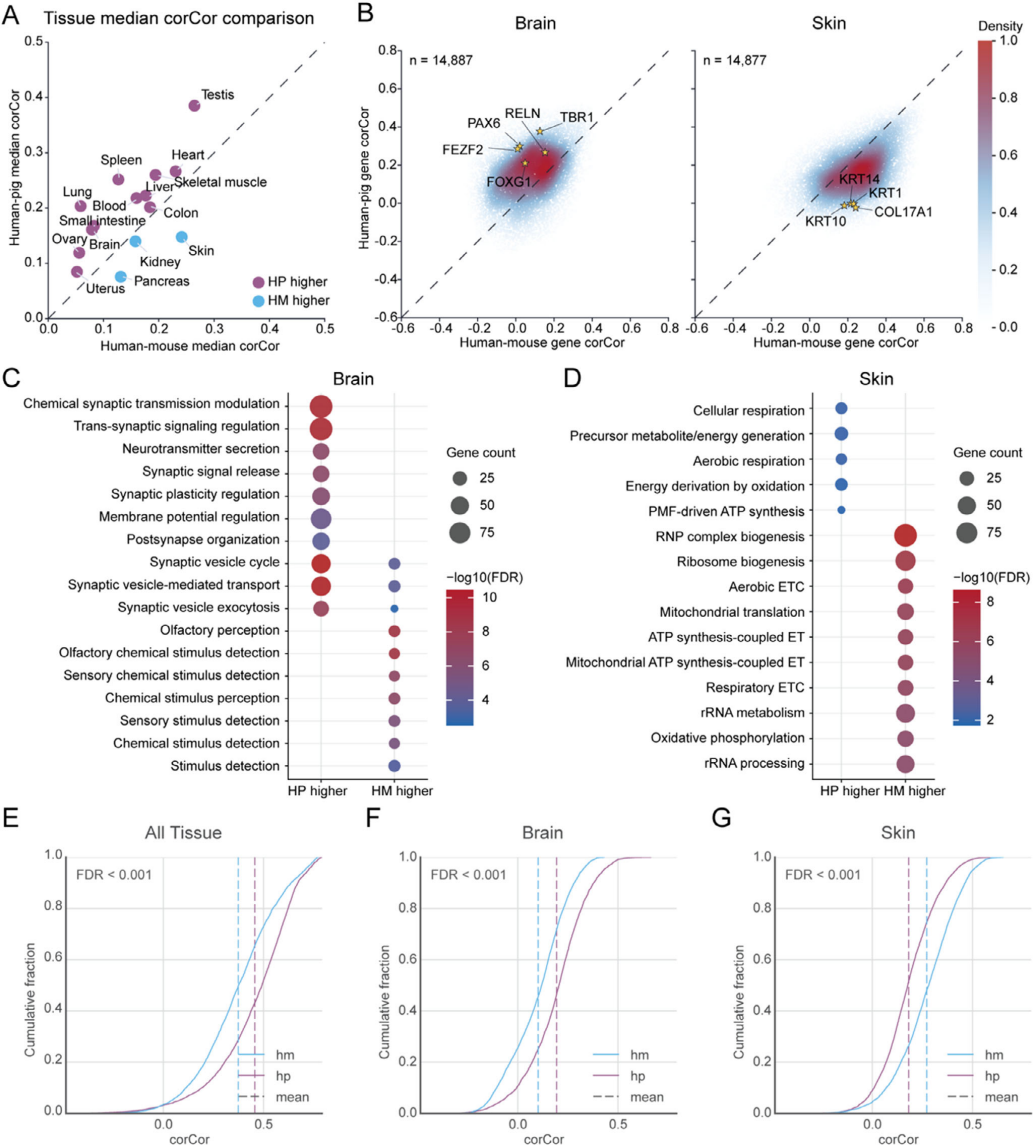
Global comparison of gene co‐expression patterns across humans, pigs, and mice. (A) Tissue‐level comparison of median corCor values across 15 matched tissues. Each point represents one tissue, with the x‐axis showing the median human‐mouse corCor and the y‐axis showing the median human‐pig corCor. (B) Gene‐level comparison of human‐mouse and human‐pig corCor values in brain and skin. Each point represents one gene and is colored by local two‐dimensional density. The dashed diagonal line indicates equal corCor values. (C) GO Biological Process enrichment analysis of human‐pig‐preferred high‐corCor genes and human‐mouse‐preferred high‐corCor genes in brain. Dot size represents the number of genes assigned to each GO term, and color indicates enrichment significance, measured as ‐log10(FDR). (D) Same as in (C), but specifically for skin tissue. (E) Cumulative distribution of gene‐level corCor values among constrained genes across all tissues. Curves show the cumulative fraction of constrained genes with corCor values less than or equal to each value on the x‐axis for human‐mouse and human‐pig comparisons within each tissue and across all tissues combined. Within each tissue, matched gene‐level corCor values were compared between human‐pig and human‐mouse comparisons using a two‐sided paired Wilcoxon signed‐rank test. *P* values were adjusted across tissues using the Benjamini‐Hochberg method. (F) Same as in (E), but specifically for brain tissue. (G) Same as in (E), but specifically for skin tissue.

To compare transcriptional regulatory similarity across species, we quantified gene co‐expression conservation using the correlation of the correlation coefficient (corCor; Methods). For each gene, corCor measures the cross‐species preservation of its gene‐centered co‐expression profile by comparing its correlation pattern with all other one‐to‐one orthologs in matched tissues (Figure S1A). Thus, a high corCor value indicates that the gene retains a similar co‐expression context across species, reflecting conservation of its regulatory associations rather than similarity in absolute expression level. Based on this indicator, we systematically compared the transcriptomic similarity among three species. The results showed that human‐pig comparisons exhibited higher corCor values than human‐mouse comparisons in 12 of 15 tissues, whereas skin, kidney, and pancreas showed higher corCor scores in human‐mouse pairs (Figure 2A; Figure S1B and Table S3), and this observation was also supported by well‐established tissue‐associated genes. For example, brain developmental and cortical organization‐associated genes, including *PAX6*, *FOXG1*, *TBR1*, *FEZF2*, and *RELN*, exhibited higher human‐pig corCor values in brain, whereas epidermal differentiation and structural genes *KRT14*, *KRT1*, *KRT10*, and *COL17A1* exhibited higher human‐mouse corCor in skin (Figure 2B). This overall pattern was reproduced in sample‐size‐matched random resampling analyses, with human‐pig comparisons showing higher corCor values across most tissues (Figure S1C).

We then used brain and skin as representative tissues to examine the biological function of species‐preferred high‐corCor genes. In brain, human‐pig‐preferred high‐corCor genes were enriched for synaptic organization, neuronal signaling, and chemical synaptic transmission, whereas human‐mouse‐preferred high‐corCor genes were mainly associated with olfactory and chemical stimulus perception (Figure 2C and Table S4). By contrast, in skin, human‐mouse‐preferred high‐corCor genes showed stronger enrichment for ribosome biogenesis, mitochondrial translation, oxidative phosphorylation, and related metabolic processes (Figure 2D and Table S4). These gene‐program‐level differences indicate that the overall tendency toward higher human‐pig co‐expression conservation is not uniform across tissues, but is shaped by tissue‐specific regulatory programs.

In addition, to independently validate this observation, we examined 3819 constrained human genes defined by gnomAD (v4) based on LOEUF score (loss‐of‐function observed/expected upper bound fraction) [29, 30] (Table S2). Compared with remaining orthologous genes, constrained genes displayed significantly higher cross‐species corCor values (Figure S1D), consistent with stronger preservation of their co‐expression relationships. Importantly, the distribution of corCor values for constrained genes recapitulated the species‐ranking observed in the complete ortholog set, with human‐pig comparisons showing higher conservation than human‐mouse comparisons across the same 12 tissues (Figure 2E–G and Figure S2). Together, these results provide additional support for stronger preservation of human‐pig co‐expression relationships than human‐mouse relationships in most tissues.

### 2.3 Cross‐Species Preservation and Tissue‐Specific Divergence of Gene Co‐Expression Network Architecture

**FIGURE 3:**
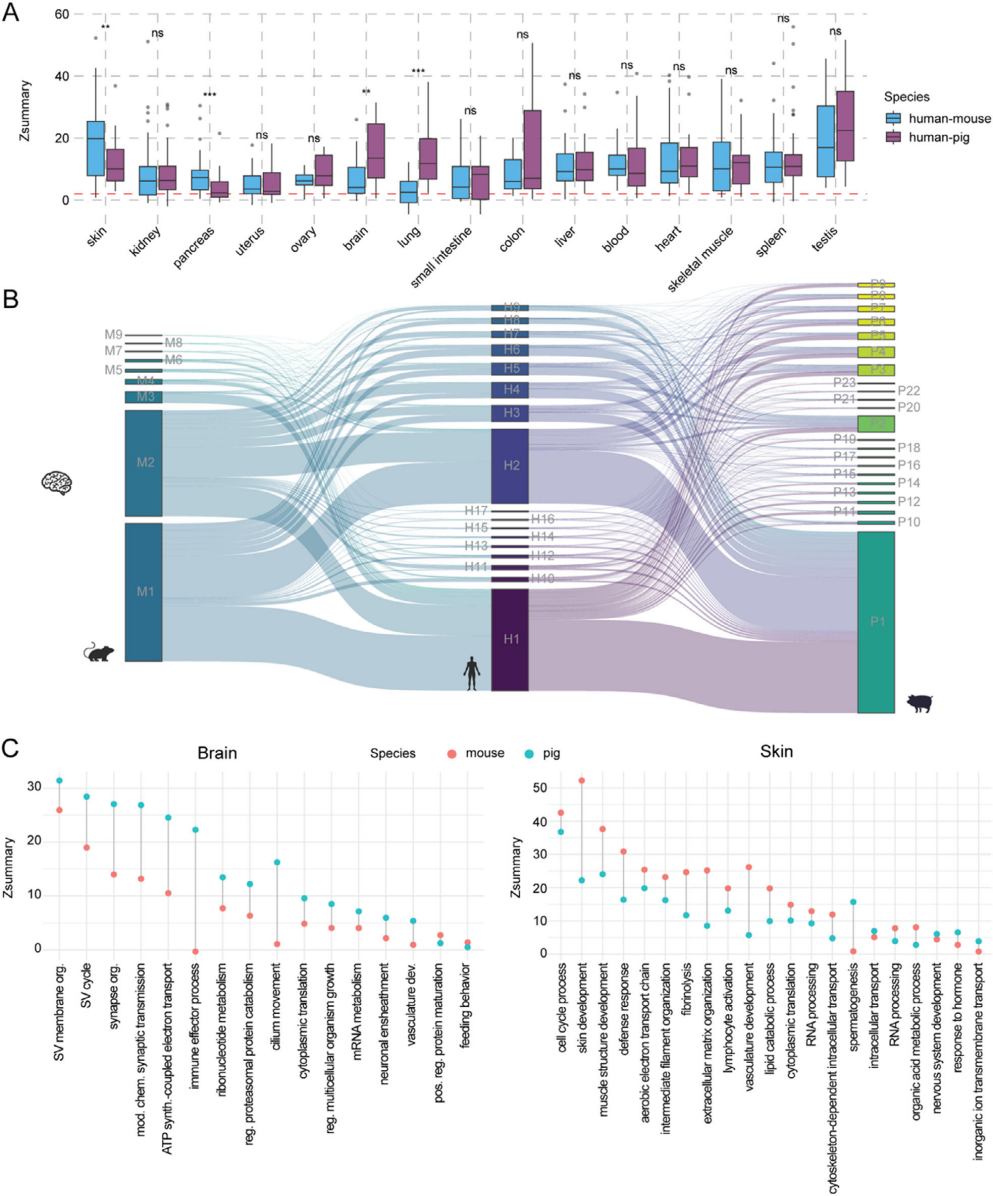
Cross‐species comparison of gene co‐expression network architecture. (A) Comparison of network module preservation. Boxplots display the distribution of module preservation statistics (*Zsummary*) for human‐mouse and human‐pig comparisons across 15 tissues, using human co‐expression modules as the reference. A horizontal red dashed line indicates *Zsummary* = 2. Within each tissue, Zsummary values for the same human modules were compared between human‐mouse and human‐pig using the two‐sided paired Wilcoxon test. Statistical significance is indicated by n.s. (non‐significant), ^*^ (*P* < 0.05), ^**^ (*P* < 0.01), ^***^ (*P* < 0.001), and ^****^ (*P* < 0.0001). (B) A three‐layer Sankey diagram summarizes the mapping of one‐to‐one orthologous genes between WGCNA co‐expression modules in mouse (left), human (middle), and pig (right). Each gene was assigned to one module in each species. Node width represents the number of genes assigned to each module, and link width represents the number of shared orthologous genes between modules in adjacent species comparisons, including mouse‐human and human‐pig. (C) Functional characterization of human co‐expression modules in brain and skin. Dot plots show representative GOBP enrichment terms for human modules in the brain and skin. Representative GO enrichment terms for each human module are displayed on the x‐axis.

We next applied WGCNA to compare higher‐order network organization among humans, pigs, and mice. Co‐expression networks were independently constructed for each tissue within species, and preservation of human modules in mouse and pig was quantified using the modulePreservation statistic (*Zsummary*). Overall, human co‐expression modules were broadly preserved in both mouse and pig comparisons, with 81.6% and 83.4% of module preservation estimates across the 15 tissues showing *Zsummary* > 2 in the human‐mouse and human‐pig comparisons, respectively (Figure 3A and Table S5). Within this overall pattern of broad preservation, the extent of module preservation varied across tissues. For example, significant differences in *Zsummary* distributions between the two comparisons were observed in the brain, skin, and pancreas, with higher median *Zsummary* values in the human‐pig comparison for brain, and higher median *Zsummary* values in the human‐mouse comparison for skin and pancreas (Figure 3A), consistent with the tissue‐dependent patterns identified by the corCor analysis.

Furthermore, human co‐expression modules were partially reorganized across species, with individual human modules often corresponding to multiple pig or mouse modules rather than maintaining simple one‐to‐one correspondence (Figure 3B and Figure S3A). Although module membership changed across species, the underlying biological functions were broadly preserved across species. For example, several human brain co‐expression modules associated with synaptic organization, synaptic vesicle organization, chemical synaptic transmission, and synaptic vesicle cycling showed strong preservation in both mouse and pig (Figure 3C). Consistently, the corresponding mouse‐human and pig‐human overlapping genes were enriched for related GOBP terms, including regulation of trans‐synaptic signaling, vesicle‐mediated transport in synapse, synaptic vesicle cycle, neurotransmitter secretion and synaptic vesicle exocytosis (Table S6). A similar pattern was also observed in skin, where module membership was reorganized across species, whereas corresponding modules retained conserved functional characteristics, including skin development and intermediate filament organization (Figure 3C and Table S6). Together, these results suggest that core tissue‐specific functional programs are broadly conserved among humans, pigs, and mice, whereas module composition can undergo cross‐species reorganization within co‐expression networks.

### 2.4 Shared Tissue‐Specific Genes Highlight Conserved Core Tissue Programs Across Species

**FIGURE 4:**
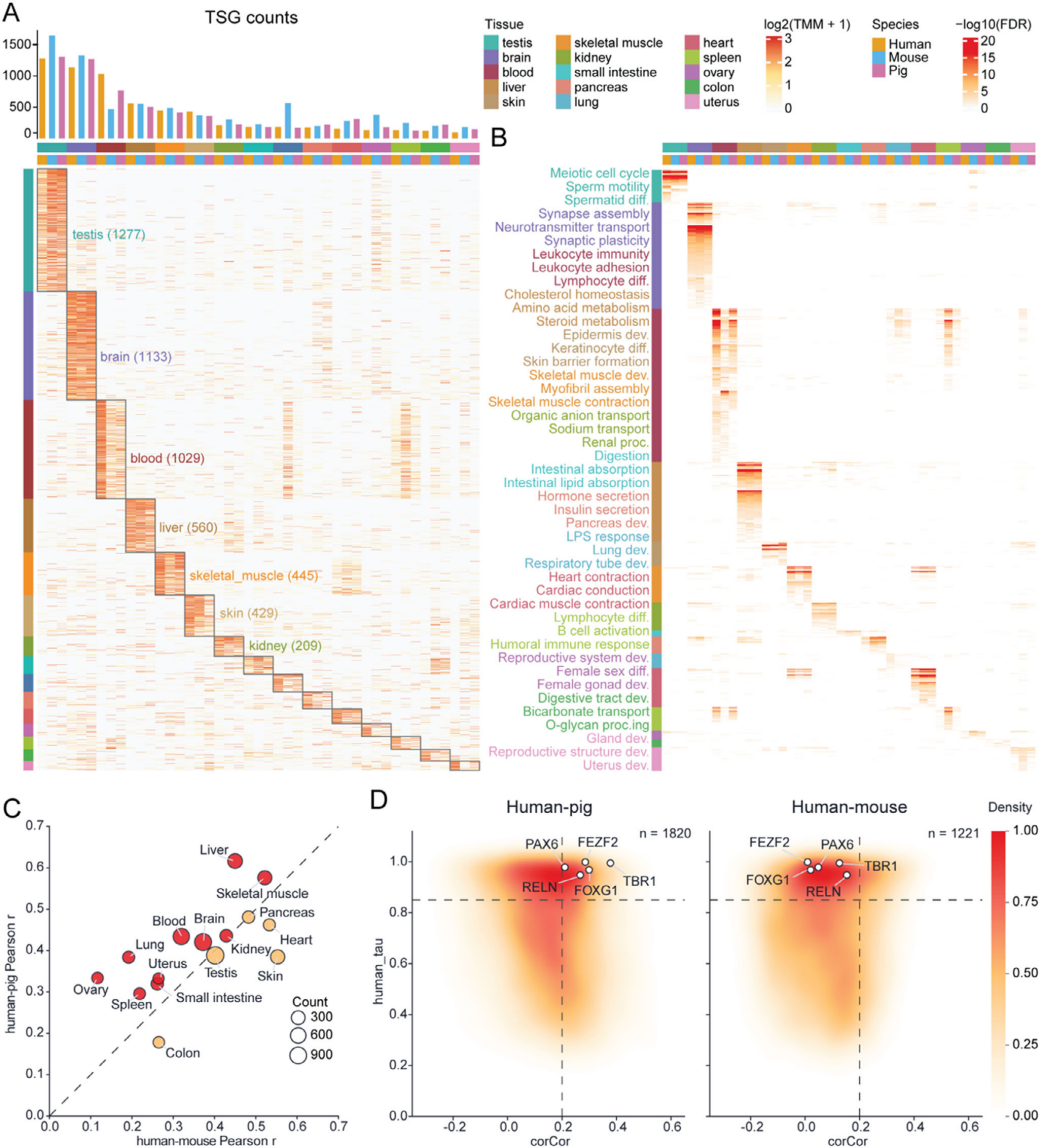
Conservation of tissue‐specific transcriptional programs across humans, mice, and pigs. (A) Cross‐species expression patterns of human‐defined TSGs. Heatmap showing normalized expression levels of human tissue‐specific genes across matched tissues in humans, mice, and pigs. (B) Functional enrichment of TSGs across tissues and species. Heatmap showing representative enriched GO Biological Process terms for TSGs, grouped by dominant tissue. Color indicates capped ‐log10(q‐value). (C) Cross‐species preservation of tissue specificity for human‐defined TSGs. Each point represents one human‐defined tissue TSG set. The x‐axis shows the Pearson correlation of τ scores between human and mouse orthologs, and the y‐axis shows the corresponding Pearson correlation between human and pig orthologs. Point size indicates the number of human‐defined TSGs in each tissue. (D) Joint distribution of human τ scores and brain corCor values for orthologs. The left and right panels show the human‐pig and human‐mouse comparisons, respectively. The x‐axis shows gene‐level corCor, and the y‐axis shows the human τ score. Dashed lines indicate τ = 0.85 and corCor = 0.2.

We next focused on tissue‐specific genes (TSGs) to assess cross‐species preservation of tissue identity. Tissue specificity was quantified using the Tau (τ) index [31], and genes with τ > 0.85 were classified as TSGs. In total, we identified 5189, 6198, and 5237 TSGs in humans, mice, and pigs, respectively, with an average of 346, 413, and 349 TSGs per tissue (Figure 4A and Table S7). These TSGs were enriched for tissue‐related biological processes consistent with their tissue annotations, such as cholesterol homeostasis in the liver, heart contraction in the heart, and synapse assembly in the brain (Figure 4B). We then compared the cross‐species similarity of tissue‐specific gene sets from two perspectives. First, we quantified the overlap between species‐specific TSG sets using the Jaccard index. In most tissues, human and pig TSG sets showed greater overlap than the corresponding human and mouse TSG sets (Figure S3B). Second, we compared the Pearson correlations between human TSGs and the corresponding mouse or pig orthologs for each tissue. Across the 15 tissues, τ‐score correlations were higher in the human‐pig comparison than in the human‐mouse comparison in 10 tissues, whereas five tissues showed equal or higher correlations in the human‐mouse comparison, further supporting the above observation by corCor assessment (Figure 4C).

We next examined the relationship between tissue‐specificity and global co‐expression conservation. Using the brain as a representative tissue, human TSGs showed a higher median corCor value in the human‐pig comparison than in the human‐mouse comparison (0.141 vs. 0.084), with more genes satisfying both thresholds (τ > 0.85 and brain corCor > 0.2) in the human‐pig comparison than in the human‐mouse comparison (1820 vs. 1221 genes, respectively; Figure 4D). Notably, several neurodevelopment and cortical organization genes, including *PAX6*, *FOXG1*, *TBR1*, *FEZF2*, and *RELN*, exhibited higher corCor values in the human‐pig comparison than in the human‐mouse comparison. This observation is consistent with the above results and previous studies highlighting the pig as a gyrencephalic large‐animal model with conserved neuroanatomical and developmental characteristics relevant to human brain research [28, 32].

### 2.5 Species‐Preferred Co‐Expression Conservation is Associated with TAD Organization

**FIGURE 5:**
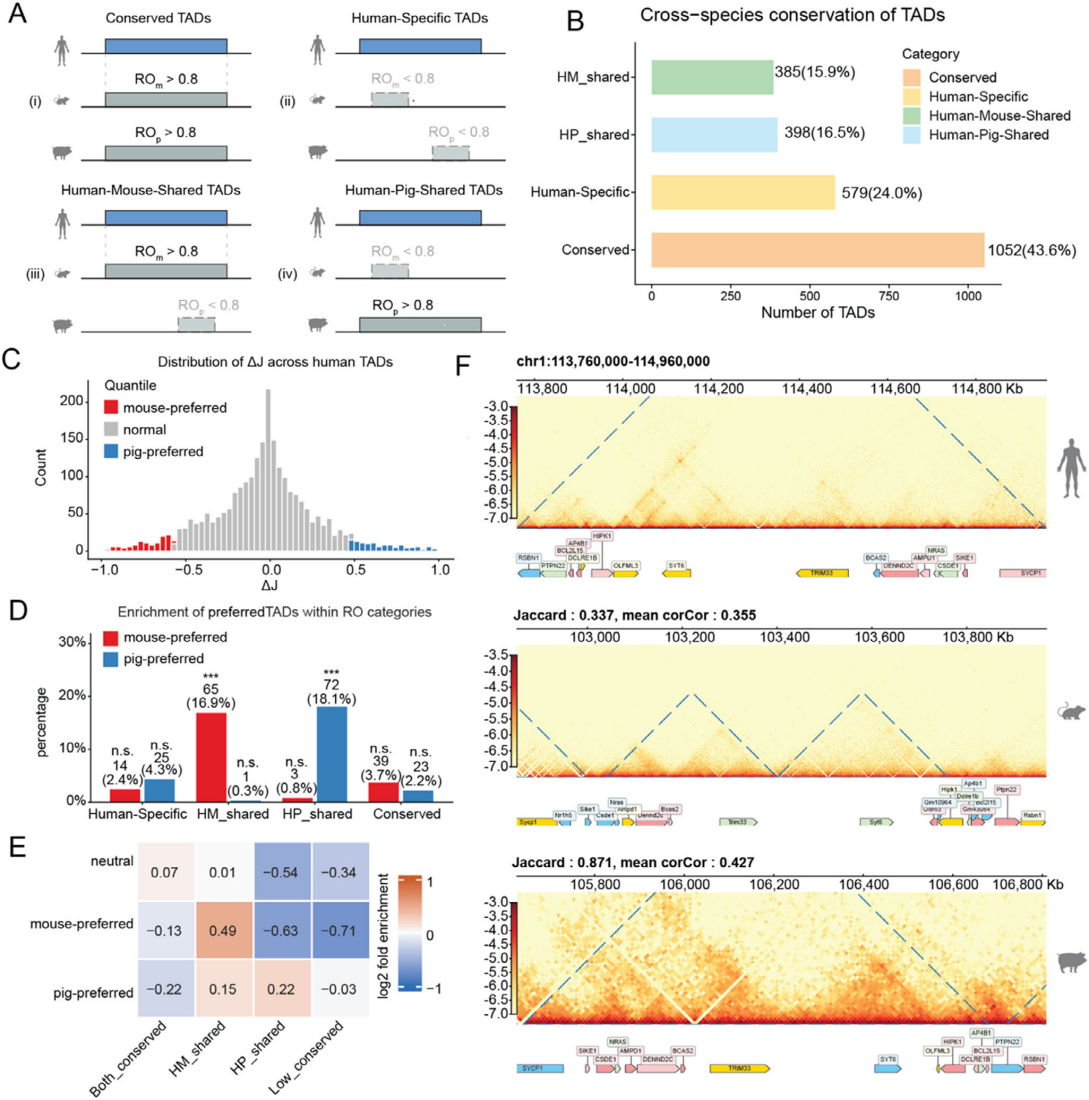
TAD similarity assessment and species‐preferred domains. (A) Classification strategy for TADs. Schematic illustrating the categorization of human TADs based on reciprocal overlap (RO) with mouse and pig domains. Categories include: Three‐way Conserved, Human‐Specific, Human‐Mouse (HM) Shared, and Human‐Pig (HP) Shared. (B) Bar plot showing the number of human TADs assigned to each evolutionary category. (C) Distribution of the continuous metric ΔJ for human TADs, defined as ΔJ = Jaccard (human, pig) – Jaccard (human, mouse). (D) Enrichment of ΔJ‐defined TADs across RO‐based TAD classes. Statistical significance was determined by Fisher's exact test and is indicated by n.s. (non‐significant) and ^***^ (FDR < 0.001). (E) Genomic Association Tester (GAT) enrichment of conservation‐defined gene categories across neutral, mouse‐preferred, and pig‐preferred TAD groups. (F) Representative visualization of chromatin contact maps, TAD boundaries, and gene annotations at a pig‐preferred TAD in human, mouse, and pig. The human genomic region chr1:113 760 000‐114 960 000 and the corresponding orthologous regions in mouse and pig are shown.

Three‐dimensional genome organization plays an important role in transcriptional regulation [33, 34, 35]. Therefore, to investigate the regulatory relationship between genomic organization and co‐expression conservation, we examined topologically associating domains (TADs) across humans, mice, and pigs. Publicly available Hi‐C‐derived TAD datasets were collected for all three species [36, 37]. Based on reciprocal overlap (RO; Methods), human TADs were classified into four categories: three‐way conserved (ROm > 0.8 and ROp > 0.8), human‐specific (ROm < 0.8 and ROp < 0.8), human‐mouse shared (ROm > 0.8 and ROp < 0.8), and human‐pig shared (ROm < 0.8 and ROp > 0.8) (Figure 5A). Three‐way conserved TADs constituted the largest fraction of human TADs (43.6%), followed by human‐specific TADs (24.0%), whereas the human‐mouse shared and human‐pig shared TADs occurred at comparable frequencies (Figure 5B). Because the RO‐based categories rely on a fixed threshold, we next adopted a continuous measure to compare TAD similarity. For each human TAD, we calculated ΔJ as the similarity indicator with pig and mouse (Methods). The resulting ΔJ score distribution was centered near zero, indicating that most human TADs showed comparable structural similarity to pig and mouse (Figure 5C). We therefore defined the top 5% and bottom 5% of ΔJ values as pig‐preferred and mouse‐preferred TADs, respectively. These TADs showed significant enrichment within the corresponding RO‐based categories: pig‐preferred TADs were enriched among human‐pig shared TADs, whereas mouse‐preferred TADs were enriched among human‐mouse shared TADs (Figure 5D). These observations were also supported by an extra validation analysis using fibroblast‐related Hi‐C datasets [38] (Figure S4).

Based on the classification of species‐preferred TADs, we next examined the role of higher‐order chromatin organization in the regulation of co‐expression conservation. We defined four corCor‐based gene sets representing distinct conservation patterns across human‐mouse and human‐pig comparisons: both‐conserved genes (n = 1971), low‐conserved genes (n = 1394), pig‐preferred genes (n = 1639), and mouse‐preferred genes (n = 1335) (Methods). Using Genomic Association Tester (GAT) [39], we assessed the enrichment of these gene categories within pig‐preferred, mouse‐preferred, and neutral TADs. Pig‐preferred genes were highly enriched in pig‐preferred TADs, whereas mouse‐preferred genes were enriched in mouse‐preferred TADs (Figure 5E), further supporting the biological relevance of corCor‐based similarity patterns and higher‐order genome architecture. A representative pig‐preferred TAD (chr1: 113 760 000‐114 960 000) showed stronger structural overlap between human and pig than between human and mouse (Jaccard score: 0.871 vs. 0.337; ΔJ = 0.534; Figure 5F). Meanwhile, genes located within this domain also exhibited higher mean corCor values in the human‐pig comparison than in the human‐mouse comparison (0.427 vs. 0.355), particularly genes involved in brain development or neuronal function, such as *SYT6*, *AP4B1*, *CSDE1*, and *TRIM33*.

### 2.6 Species‐Preferred Co‐Expression Conservation is Associated with Human Complex‐Trait Genetic Architecture

We performed stratified LD‐score regression (S‐LDSC) and MAGMA analyses across 176 human traits to assess the genetic architecture of the above four gene categories. For S‐LDSC, SNP annotations were generated by intersecting ENCODE regulatory elements (promoters and enhancers) surrounding genes in each category (Figure S5, Tables S8 and S9).

**FIGURE 6:**
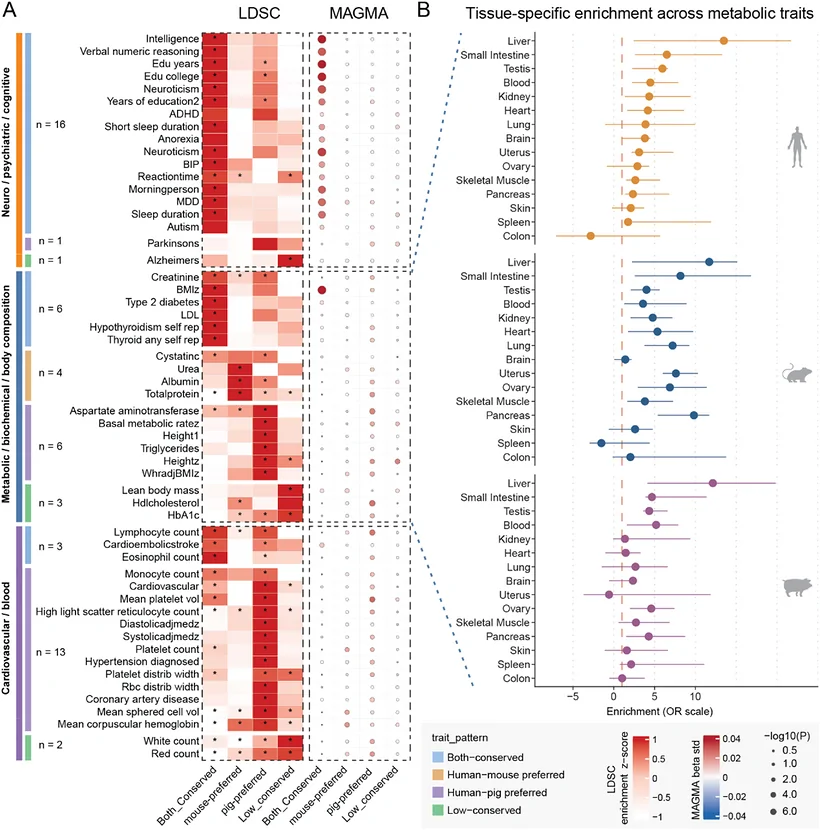
Heritability enrichment of 52 representative traits. (A) Enrichment analyses of conservation‐defined gene categories across 52 representative human complex traits. The left panel shows S‐LDSC heritability enrichment based on enhancer‐derived SNP annotations for both‐conserved, mouse‐preferred, pig‐preferred, and low‐conserved gene categories. The right panel shows MAGMA gene‐set enrichment analysis for the same conservation‐defined gene categories and trait order. Heatmap colors indicate row‐scaled LDSC enrichment in the left panel and standardized MAGMA effect size in the right panel; point size in the MAGMA panel indicates ‐log10(P‐value). (B) Metabolic‐trait‐focused S‐LDSC analysis of tissue‐specific enhancer annotations. Points show the median LDSC enrichment across 19 metabolic traits for each tissue and species, horizontal bars show bootstrap 95% confidence intervals, and the dashed vertical line marks enrichment = 1. Tissues are ordered by human median enrichment.

Enhancer‐based S‐LDSC revealed distinct heritability enrichment profiles across four categories (Figure 6A) in 52 representative traits. Both‐conserved genes exhibited the strongest and broadest enrichment across traits, with prominent signals for neuropsychiatric, cognitive, and behavioral phenotypes. In contrast, pig‐preferred genes showed higher enrichment mainly in metabolic, cardiovascular, and blood‐related traits. Mouse‐preferred and low‐conserved genes generally displayed weaker and less consistent enrichment patterns. In addition, MAGMA gene‐set analysis yielded concordant results (Figure 6A and Table S10), further supporting this observation. Together, these findings indicate that different gene sets of cross‐species co‐expression conservation are linked to distinct classes of human complex traits. In particular, pig‐preferred genes showed stronger enrichment than mouse‐preferred genes for metabolic, cardiovascular, and blood‐related traits.

To further investigate the metabolic enrichment associated with tissue‐specific regulatory programs, we generated enhancer‐based annotations from tissue‐specific genes and evaluated their heritability enrichment across 19 metabolic traits (Figure 6B and Figure S6). In humans, tissue‐specific annotations revealed a hierarchical pattern of enrichment, with liver showing the strongest signal, followed by metabolically relevant tissues including the small intestine and kidney. Similar tissue‐ranking patterns were observed in both pigs and mice. Notably, pig liver‐specific enhancer annotations exhibited stronger heritability enrichment than mouse, consistent with the preferential enrichment of metabolic traits among pig‐preferred genes. These results suggest that the overall tissue regulatory framework underlying metabolic trait heritability is broadly conserved across mammals, while pig liver‐associated regulatory annotations show closer correspondence to the genetic architecture of human metabolic traits than the corresponding mouse annotations.

### 2.7 An Interactive Portal Enables Integrative Gene‐Centered Exploration Across Humans, Pigs, and Mice

Given the context‐dependent patterns of cross‐species similarity observed across regulatory layers, we developed an interactive portal to support gene‐centered evaluation of human‐mouse and human‐pig correspondence across tissues. The portal integrates five analytical layers, including gene‐level co‐expression similarity, co‐expression module assignment, tissue specificity, 3D chromatin‐domain correspondence, and trait or phenotype annotations. We used *CRH* (corticotropin‐releasing hormone), a key regulator of the hypothalamic‐pituitary‐adrenal (HPA) axis [40], as an illustrative example (Figures S7–S9).

Tissue specificity analysis shows that *CRH* retains brain‐specific expression across humans, mice, and pigs. At the gene‐level co‐expression layer, human *CRH* shows higher corCor with pig than with mouse (0.304 vs. 0.065). Consistently, chromatin‐domain analysis showed greater correspondence between the human and pig *CRH* loci than between the human and mouse loci. By contrast, module‐level analysis showed a different pattern. Although *CRH* was assigned to co‐expression modules in all three species, the overlap between the human CRH‐containing module and its orthologous counterpart was higher for mouse than for pig (0.399 versus 0.011). This discrepancy indicates that cross‐species similarity for the same gene can differ between local co‐expression structure, chromatin‐domain organization, and broader module‐level network context. The portal further connects these regulatory comparisons with trait and phenotype annotations. In the resources integrated for this analysis, *CRH* was linked to one direct human gene‐trait association from the GWAS Catalog, 40 mouse phenotype annotations from MGI, and 50 pig gene‐based GWAS records from PigBiobank. These species‐specific records provide complementary phenotypic context for evaluating whether the observed regulatory similarity is relevant to the biological question under consideration.

Overall, this example illustrates how the portal extends the multi‐layer comparative framework to individual genes and tissues. Rather than supporting a single species‐level ranking of animal model similarity, these results highlight the need to evaluate model suitability in a gene‐, tissue‐, regulatory‐layer‐, and phenotype‐specific manner.

## 3 Discussion

In summary, this study assembled a large‐scale tissue‐matched transcriptomic resource comprising more than 16000 RNA‐seq samples from humans, pigs, and mice and established a unified framework for evaluating cross‐species similarity across multiple molecular layers. By integrating global co‐expression conservation, co‐expression network architecture, tissue‐specific gene programs, chromatin‐domain organization, and human complex‐trait genetics, we generated a comprehensive view of how transcriptional programs are conserved and diverge across humans, pigs, and mice. In addition, this study provides an analytical framework for evaluating animal models through transcriptomic and regulatory conservation. The accompanying interactive portal further enables gene‐centered exploration of cross‐species similarity and provides a practical resource for selecting appropriate animal models for specific biological questions.

A central finding of this study is that human‐pig comparisons generally showed stronger co‐expression similarity than human‐mouse comparisons across many tissues, although this pattern was not universal. In particular, skin, kidney, and pancreas showed stronger human‐mouse similarity, indicating that cross‐species co‐expression similarity varies by tissue rather than following a uniform species‐level pattern. By extending the comparison beyond expression abundance to co‐expression relationships, network architecture, tissue‐specific programs, and chromatin‐domain organization, our analyses indicate that cross‐species similarity is layered and context‐dependent. The concordance between species‐preferred co‐expression profiles and species‐preferred TAD conservation further suggests that directional transcriptional conservation is associated with higher‐order genome organization. Integration with human complex‐trait genetics also showed that different conservation patterns are linked to distinct phenotype domains, with pig‐preferred genes more strongly associated with metabolic, cardiovascular, biochemical, and blood‐related traits, whereas both‐conserved genes showed broader enrichment for neuropsychiatric, cognitive, and behavioral phenotypes. Together, these findings support a context‐dependent view of model selection, in which the suitability of pigs or mice should be evaluated according to the specific tissue, gene, regulatory layer, and biological question of interest.

Although this study provides a multi‐layer comparative framework and an interactive resource for evaluating human‐mouse and human‐pig regulatory correspondence across tissues, several limitations should be noted. First, our analyses were restricted to one‐to‐one orthologous protein‐coding genes, which ensures direct cross‐species comparability but does not capture species‐specific genes, duplicated genes, non‐coding RNAs, or more complex orthologous relationships. Thus, the conclusions primarily reflect conservation and divergence within the shared orthologous regulatory landscape. Second, the cross‐species TAD analysis relied on publicly available Hi‐C datasets that were not fully matched for tissue or cell type across species. Therefore, some species‐specific domains may be influenced by differences in cellular context. Third, the S‐LDSC and MAGMA analyses associate conservation‐defined regulatory annotations with human complex‐trait genetic architecture, but they do not establish causal links between molecular conservation and disease model performance. Experimental validation in disease‐ or perturbation‐specific contexts will be required to determine how these molecular similarities translate into practical model selection.

As cross‐species functional genomics resources continue to expand, comparative analyses can move beyond global assessments of species similarity toward more precise evaluations of biological context. Our results suggest that transcriptome comparison is highly context‐dependent across tissues, genes, regulatory networks, chromatin structures, and trait domains. Therefore, no single model organism should be assumed to be universally optimal for all biological questions. Rather than asking whether pigs or mice are globally better surrogates for humans, a more informative question is when, where, and for which biological process each model provides the closest representation of human biology. Future integration of single‐cell transcriptomic, epigenomic, and three‐dimensional genome datasets across species will further refine this context‐aware framework and improve context‐informed selection of animal models in functional genomics and translational research.

## 4 Conclusions

Collectively, we established a large‐scale, tissue‐matched transcriptomic resource across humans, pigs, and mice and developed a unified, multi‐layer framework to evaluate cross‐species similarity in transcriptional regulation. By integrating global co‐expression similarity, co‐expression network architecture, tissue‐specific gene programs, higher‐order genome organization, and associations with human complex traits, we show that cross‐species similarity is not a single global property, but a layered and context‐dependent feature. Although pigs generally showed stronger transcriptomic correspondence to humans than mice across most tissues at the global co‐expression level, this pattern was not consistent across all tissues, regulatory layers, or phenotypic domains. Altogether, these findings support a context‐aware view of model selection, in which the relative suitability of pigs and mice depends on the specific tissue, regulatory dimension, and biological question under investigation. Our study therefore provides both a comparative framework and a resource for evaluating when and where each model may better represent particular aspects of human biology and disease.

## 5 Experimental Section

### 5.1 RNA‐Seq Data Collection, Preprocessing, and Normalization

Human RNA‐seq data were obtained from the Genotype‐Tissue Expression (GTEx) project (v8), using the consortium's uniformly processed quantification files [41]. For mouse and pig, RNA‐seq datasets were collected from the IAnimal database [14]. All raw sequencing reads were downloaded using SRAToolkit [42], and adapters and low‐quality bases were removed using fastp (v0.23.4) [43]. Samples with low sequencing depth (<6 million reads) were excluded. High‐quality reads were aligned to the respective reference genomes (pig: Sscrofa11.1; mouse: GRCm39) using HISAT2 [44]. Transcript assembly and gene‐level quantification were performed using StringTie (v2.1.7) [45], yielding Transcripts Per Million (TPM) values. For cross‐species comparisons, we retained only protein‐coding genes with one‐to‐one orthologs across human (GRCh38.p13), mouse (GRCm39), and pig (Sscrofa11.1), resulting in a final set of 14910 orthologs (Table S2).

To improve comparability across samples after restricting the analysis to one‐to‐one orthologous protein‐coding genes, we adapted the TMM‐based normalization strategy used in the Human Protein Atlas Blood Atlas framework for cross‐species analysis [46]. In brief, TPM‐derived expression matrices were normalized separately within each species to reduce sample‐level compositional biases. TMM normalization factors were estimated using ‘edgeR::calcNormFactors’ [47], and normalized expression values were obtained with ‘edgeR::cpm’ using TMM‐adjusted effective library sizes. The normalized expression values were transformed as log2(TMM + 1) for downstream comparative analyses.

### 5.2 Cross‐Species UMAP Visualization of Tissue Transcriptomes

To visualize the global organization of cross‐species tissue transcriptomes, expression profiles from humans, pigs, and mice were restricted to 14910 one‐to‐one orthologous protein‐coding genes shared across the three species. TMM values were transformed as log2(TMM + 1), followed by gene‐wise z‐score normalization within each species. The resulting expression matrices were merged into a unified sample‐by‐gene matrix comprising 16 085 samples.

The merged matrix was first reduced by principal component analysis. The first 12 principal components were then used as input for two‐dimensional UMAP [48] embedding with Euclidean distance, 40 nearest neighbors, and a minimum distance of 0.25. The same UMAP embedding was visualized with samples colored by tissue identity or species to assess whether the major transcriptomic structure was associated primarily with tissue identity or species.

### 5.3 Quantification of Co‐Expression Conservation (corCor)

Gene‐centered co‐expression conservation across species was quantified using the correlation of the correlation coefficient (corCor) [49]. For each tissue, expression profiles of the shared one‐to‐one orthologous genes were used to calculate within‐species gene‐gene Pearson correlation matrices. For a given gene (g) among (N) one‐to‐one orthologs, its correlations with all other (N‐1) orthologs were extracted as a gene‐centered co‐expression vector *V_g, species_*. The corresponding vector *V_g’, species'_* was generated for its ortholog (g') in the comparison species, with the same ortholog order maintained between vectors. The Pearson correlation between *V_g, species_* and *V_g’, species'_* was defined as the corCor value. Higher corCor values indicate greater preservation of the gene‐centered co‐expression profile, reflecting a more similar co‐expression neighborhood across species.

To assess whether gene‐level co‐expression conservation was associated with human genetic constraint, we integrated gene constraint metrics from gnomAD v4.0 [29, 30]. The gnomAD constraint metrics were mapped to human genes in the high‐confidence one‐to‐one orthologous gene set shared among humans, pigs, and mice. Genes with a LOEUF < 0.6 were classified as constrained, yielding 3819 constrained genes within the orthologous gene set (Table S2). For each tissue and species comparison, the distributions of corCor values were compared between constrained genes and the remaining orthologous genes using a two‐sided Wilcoxon rank‐sum test. Human‐pig and human‐mouse corCor values for constrained genes were further compared using a two‐sided paired Wilcoxon signed‐rank test.

### 5.4 Weighted Gene Co‐Expression Network Analysis (WGCNA)

To delineate the architectural conservation and species‐specific differences in co‐expression module organization, we independently constructed co‐expression networks for human, mouse, and pig utilizing the “blockwiseModules” function from the WGCNA R package (v1.72‐1) [50]. Normalized expression values (one‐to‐one orthologs) were log‐transformed prior to network construction. For each dataset, the soft‐thresholding power (β) was optimized to approximate scale‐free topology (*R^2^* > 0.85). Key parameters were set as follows: TOMType = “unsigned”, minModuleSize = 30, and mergeCutHeight = 0.25. Genes were hierarchically clustered and assigned to distinct modules labeled by colors.

To quantitatively assess cross‐species preservation and differences in co‐expression module organization, we computed module preservation statistics using the “modulePreservation” function. Using the human networks as the reference, this approach allowed us to evaluate the extent to which human co‐expression modules were preserved in mouse and pig. Following established thresholds [51], *Zsummary* > 10 indicated highly preserved modules, 2 < *Zsummary* ≤ 10 moderately preserved modules, and *Zsummary* ≤ 2 non‐preserved modules. Differences in these module preservation scores (*Zsummary*) between human‐mouse and human‐pig pairs across all 15 tissues were statistically evaluated utilizing the two‐sided paired Wilcoxon test.

### 5.5 Tissue‐Specific Expression Analysis

We systematically quantified the tissue specificity of gene expression utilizing the Tau index, a highly robust metric for identifying tissue‐defining genes originally described by Yanai et al. [31]. Tissue‐specificity was quantified for each gene using the tissue‐specificity index (τ), defined as:

$$\tau =\frac{\sum_{i=1}^{N}\left(1-\frac{x_{i}}{x_{\max}}\right)}{N-1}$$

where *x_i_* is the expression of a gene in tissue *i*, *x*_max_ is its maximum expression across tissues, and *N* is the total number of tissues (N  =  15). Genes with τ > 0.85 were classified as tissue‐specific genes (TSGs).

### 5.6 Comparative Analysis of 3D Genome Organization

To delineate cross‐species structural conservation, publicly available Hi‐C‐derived TAD datasets were collected for all three species, including human, mouse [36], and pig datasets [37]. TAD intervals from the mouse (mm10) and pig (susScr11.1) genomes were projected onto the human reference genome (hg38) using the UCSC LiftOver utility [52] with a minimum sequence match threshold of 0.1 to improve recovery of cross‐species syntenic TAD intervals while avoiding excessive loss of informative domains. Pairwise overlaps between human TADs and lifted‐over mouse or pig TADs were then calculated using BEDTools [53]. For each human TAD, the orthologous domain in the query species (mouse or pig) with the maximum overlap length was retained as the best hit. We then calculated conservation scores based on sequence coverage: RO_p_ (fraction of human TAD covered by pig TAD) and RO_m_ (fraction covered by mouse TAD). Human TADs were stratified into four evolutionary categories based on these thresholds:

- Three‐way conserved TADs: RO_p_ > 0.8 and RO_m_ > 0.8;
- Human‐specific TADs: RO_p_ < 0.8 and RO_m_ < 0.8;
- HM‐shared TADs: RO_p_ < 0.8 and RO_m_ > 0.8;
- HP‐shared TADs: RO_p_ > 0.8 and RO_m_ < 0.8.

Because RO‐based categories depend on a fixed cutoff, we additionally quantified similarity using a continuous Jaccard index for each best‐hit pair:

$$J\left(A,B\right)=\frac{\left|A\cap B\right|}{\left|A\cup B\right|}=\frac{\mathrm{overlap}\_\mathrm{bp}}{\mathrm{lenA}+\mathrm{lenB}-\mathrm{overlap}\_\mathrm{bp}}$$

We then defined the relative lineage‐bias metric:

$$\Delta J=J_{HP}-J_{HM}$$

Positive ΔJ values indicate stronger human‐pig structural similarity, and negative values indicate stronger human‐mouse structural similarity. Human TADs in the top 5% of ΔJ were defined as pig‐preferred TADs, whereas those in the bottom 5% were defined as mouse‐preferred TADs.

To evaluate the robustness of the cross‐species TAD comparison to the choice of Hi‐C datasets, we performed an additional validation analysis using the most closely comparable fibroblast‐related Hi‐C datasets available across the three species. Processed TAD files were obtained from GSE167579 [38], including human skin fibroblasts, pig embryonic fibroblasts, and mouse embryonic fibroblasts. The validation analysis was performed using the same analytical framework as the primary TAD analysis. Briefly, mouse and pig TAD intervals were projected onto the human reference genome using UCSC LiftOver with the same minimum sequence match threshold, and best‐hit overlaps between human TADs and lifted‐over mouse or pig TADs were identified using BEDTools. RO‐based TAD classification, Jaccard index calculation, ΔJ estimation, and the classification of pig‐preferred and mouse‐preferred TADs were then performed using the same criteria as described above.

### 5.7 Classification of corCor‐Based gene Conservation categories

Gene‐level co‐expression conservation was quantified using corCor values for human‐mouse and human‐pig comparisons. Genes with valid corCor values in both comparisons were retained for category assignment. To improve comparability between the two comparisons and reduce the influence of correlation‐scale nonlinearity, corCor values were transformed using Fisher's Z transformation. The transformed values were denoted as *Z_hm_* for the human‐mouse comparison and *Z_hp_* for the human‐pig comparison. Directional differences in co‐expression conservation were quantified as ΔZ = *Z_hm_*− *Z_hp_* where negative values indicate stronger co‐expression similarity in the human‐pig comparison and positive values indicate stronger similarity in the human‐mouse comparison.

Genes were assigned to corCor‐based conservation categories using a hierarchical quantile‐based strategy. Quantile thresholds were calculated from the distributions of *Z_hm_*, *Z_hp_*, and ΔZ across all genes retained for corCor‐based classification. Genes with high co‐expression similarity in both comparisons were first classified as both‐conserved genes, defined as *Z_hm_* > Q_80_ and *Z_hp_* > Q_80_. Genes with low co‐expression similarity in both comparisons were classified as low‐conserved genes, defined as *Z_hm_* < Q_20_ and *Z_hp_* < Q_20_. Among the remaining genes, directionally preferred categories were defined based on both directional differences and the corresponding species‐pair corCor level. Pig‐preferred genes were defined as genes with stronger co‐expression similarity in the human‐pig comparison, using the criteria ΔZ < Q_20_ and *Z_hp_* > Q_60_. Mouse‐preferred genes were defined as genes with stronger co‐expression similarity in the human‐mouse comparison, using the criteria ΔZ > Q_80_ and *Z_hm_* > Q_60_. Genes that did not satisfy any of these criteria were retained as unclassified and were not included as focal categories in downstream enrichment analyses.

To assess whether corCor‐based gene conservation categories were associated with species‐preferred chromatin organization, we tested the enrichment of both‐conserved, low‐conserved, pig‐preferred, and mouse‐preferred genes within different TAD classes using Genomic Association Tester (GAT) [39]. Gene intervals were intersected with pig‐preferred, mouse‐preferred, and neutral TADs, and enrichment was evaluated against the genomic background of genes included in the corCor analysis. Enrichment significance was estimated using the GAT randomization framework, which compares the observed overlap between each gene category and TAD class with overlaps expected under randomized genomic intervals while preserving the defined genomic workspace.

### 5.8 Heritability Enrichment Analysis Using Stratified LD Score Regression (S‐LDSC) and MAGMA Gene‐Set Analysis

To link the above categories to human complex‐trait heritability, we focused on two complementary analyses: binary SNP‐level annotations for stratified LD score regression (S‐LDSC) [54] and MAGMA gene‐set analysis [55]. For S‐LDSC, genes of each gene category were extended by 100 kb upstream and downstream from the gene body, and the resulting regions were intersected with ENCODE enhancer or promoter annotations to generate enhancer‐ and promoter‐SNP annotations, respectively. For each category‐specific regulatory interval set, binary SNP‐level annotations were generated by mapping the intervals to SNPs in the 1000 Genomes Phase 3 European reference panel; SNPs overlapping the corresponding intervals were assigned an annotation value of 1, whereas reference SNPs outside the intervals were assigned a value of 0. We then applied S‐LDSC (v1.0.1) to partition SNP‐based heritability (h^2^) across 176 human complex traits using GWAS summary statistics from the Zenodo repository (https://zenodo.org/records/7768714). Custom LD scores for these annotations were computed using the 1000 Genomes Phase 3 European reference panel and HapMap3 SNPs excluding the MHC region, and all analyses were conditioned on the baseline‐LD v2.2 model to control for known functional annotations and LD‐related genomic features.

To complement the SNP‐level S‐LDSC analysis, we performed gene‐based and gene‐set analyses using MAGMA [55]. GWAS summary statistics for the same 176 traits were first harmonized to SNP identifier, P value, and sample‐size columns. When P values were not directly available, they were derived from χ^2^ statistics or two‐sided Z scores. SNPs were mapped to protein‐coding genes annotated in GENCODE v19 (GRCh37) using the 1000 Genomes European reference panel, with a ±100 kb window around each gene. MAGMA gene‐based analysis was then performed to compute gene‐level association statistics from SNP‐level GWAS P values while accounting for linkage disequilibrium among SNPs using the 1000 Genomes European reference panel.

### 5.9 Functional Annotation of Animal Genomes

To establish a standardized functional baseline across all evaluated species, the genomic coordinates, primary sequences, structural architectures, and foundational metadata for all annotated genes were systematically extracted from the Ensembl database (Release 104) [56]. Because divergent species‐specific annotation pipelines can introduce analytical artifacts, we unified the functional annotation standards to ensure cross‐species comparisons. Specifically, InterProScan (v5.27) [57] was deployed to identify conserved protein domains and strictly map them to standardized Gene Ontology (GO) terms [58].

## Author Contributions

J.W.D. conceived the study, performed the data analysis, and wrote the original draft. Y.L., X.S., X.H., Z.S.T., G.W.Y., and H.J.Z. performed bioinformatics analyses and methodology implementation. J.Y.X., H.L., Y.W., K.K.L., J.L., X.Z., C.Y.L., and S.J.W. contributed to comprehensive data curation, validation, and quality control. L.L.F., L.L.Y., X.Y.L., S.H.Z., and X.L.L. provided a critical review of the manuscript. J.J.L. co‐designed the study and revised the manuscript. Y.H.F. supervised the project, acquired funding, and critically reviewed and edited the final manuscript. All authors read and approved the final manuscript.

## Conflicts of Interest

The authors declare no conflicts of interest.

## Supporting information




**Supporting File 1**: advs77881‐sup‐0001‐SuppMat.docx.

**Supporting File 2**: advs77881‐sup‐0002‐SuppMat.xlsx.

## Data Availability

Human RNA‐seq data were obtained from the Genotype‐Tissue Expression (GTEx) project (v8) via the GTEx Portal (https://gtexportal.org). Pig and mouse public RNA‐seq datasets utilized in this study were systematically curated from the IAnimal database (https://ianimal.pro). The corresponding TPM‐normalized gene expression matrices are available through the IAnimal download page. The interactive cross‐species gene query and visualization portal developed in this study is publicly accessible at https://ianimal.pro/tools/gallery/cross‐species‐comparison. To ensure full computational reproducibility, all custom scripts, analytical pipelines, and processed data matrices generated during this study have been deposited in GitHub and are freely available at https://github.com/Jingwen‐d/cross‐species‐model‐analysis.
