# Supplementary material for: Multi‐Layer Transcriptomic Comparison Across Human, Pig, and Mouse Informs Tissue‐ and Trait‐Specific Animal Model Selection
Source: Adv Sci (Weinh). 2026 Sep 27:e77881. Online ahead of print. doi: 10.1002/advs.77881 (PMC13616397; doi:10.1002/advs.77881)
Supplement: Supplementary file 1 [file ADVS-9999-e77881-s001.docx]

**Figure S1. Global comparison of gene co-expression patterns across humans, pigs, and mice.** (A) Schematic overview of gene-centered co-expression conservation quantified by corCor. (B) Comparison of co-expression corCor distributions. Violin plots showing the distribution of correlation of correlations (corCor) values for human-mouse and human-pig pairs across 15 tissues. Significance was assessed using two-sided Wilcoxon test (*** P < 0.001). (C) Random downsampling analysis of tissue-level corCor patterns. For each matched tissue and species comparison, samples from the species with the larger sample size were randomly downsampled to match the sample size of the smaller species. This procedure was repeated 30 times. In each round, the median gene-level corCor value was calculated separately for human-pig and human-mouse comparisons, and ΔcorCor was defined as median corCor(human-pig) minus median corCor(human-mouse). Colored points indicate the mean ΔcorCor across 30 downsampling rounds, and horizontal bars represent the empirical 95% interval, defined by the 2.5th and 97.5th percentiles. Black vertical ticks indicate ΔcorCor calculated from the full TMM-normalized dataset without downsampling. The dashed vertical line marks ΔcorCor = 0. Positive values indicate higher human-pig corCor, whereas negative values indicate higher human-mouse corCor. (D) Distribution of gene-level co-expression conservation for constrained and non-constrained genes across tissues. Boxplots compare human-mouse and human-pig corCor values between constrained and non-constrained genes across all analyzed tissues. Constrained genes were defined using gnomAD constraint criteria, whereas non-constrained genes comprised all remaining one-to-one orthologous genes not included in the constrained set. For each species comparison, a one-sided Mann-Whitney U test was used to test whether constrained genes exhibited higher corCor values than non-constrained genes. Statistical significance is indicated by **** *P* < 0.0001.

**Figure S2. Validation analyses of gene-level co-expression conservation. (**A-M) Cumulative distribution of gene-level corCor values for constrained genes across tissues. Curves show the distribution of gene-level corCor values for constrained genes in human-mouse and human-pig comparisons within each tissue. Human-mouse comparisons are shown in blue, and human-pig comparisons are shown in purple. Dashed vertical lines indicate the mean corCor value for each comparison. Within each tissue, matched gene-level corCor values were compared between human-pig and human-mouse comparisons using a two-sided paired Wilcoxon signed-rank test. *P* values were adjusted across tissues using the Benjamini-Hochberg method. Tissue-specific ECDF curves are shown for liver (A), lung (B), colon (C), pancreas (D), spleen (E), testis (F), ovary (G), kidney (H), skeletal muscle (I), blood (J), small intestine (K), uterus (L), and heart (M).

**Figure S3. Cross-species comparison of WGCNA module organization and tissue-specific gene overlap.** (A) Three-layer Sankey diagram summarizing cross-species overlap of WGCNA module membership for one-to-one orthologs in skin, analogous to the brain example in Figure 3B. Node width represents module gene counts and edge width denotes the number of shared orthologs between adjacent species (mouse→human and human→pig). (B) Jaccard overlap of tissue-matched TSG sets across species, comparing human-pig versus human-mouse pairs across tissues.

**Figure S4.** **TAD similarity assessment and species-preferred domains**. (A) Classification strategy for TADs. Schematic illustrating the categorization of human TADs based on reciprocal overlap (RO) with mouse and pig domains. Categories include: Three-way Conserved, Human-Specific, Human-Mouse (HM) Shared, and Human-Pig (HP) Shared. (B) Bar plot showing the number of human TADs assigned to each evolutionary category. (C) Distribution of the continuous metric ΔJ for human TADs, defined as ΔJ = Jaccard (human, pig) - Jaccard (human, mouse). (D) Enrichment of ΔJ-defined TADs across RO-based TAD classes. Statistical significance was determined by Fisher’s exact test and is indicated by n.s. (non-significant) and *** (FDR < 0.001).

**Figure S5. Enhancer- and promoter-based S-LDSC analyses reveal distinct heritability enrichment patterns across human complex traits.** (A) S-LDSC heritability enrichment analysis based on enhancer-linked annotations for conservation-defined gene categories across 176 human complex traits. (B) S-LDSC heritability enrichment analysis based on promoter-linked annotations for conservation-defined gene categories across 176 human complex traits.

**Figure S6. The heatmap shows LDSC enrichment of TSG enhancers for each metabolic GWAS trait and tissue combination in human, mouse, and pig.** Each tile represents the LDSC enrichment value for one trait-tissue pair, with colors ranging from blue to red indicating lower to higher enrichment and white corresponding to enrichment = 1. Black dots denote trait-tissue pairs with enrichment > 1 and nominal significance at P < 0.05. The color scale was capped at -10 and 40 to reduce the visual influence of unstable outlier enrichment estimates.

**Figure S7. Overview of the portal homepage.** The query interface supports searches by gene symbol/gene ID, tissue, and Tau threshold. The homepage also presents the overall analytical framework of the portal, integrating global co-expression conservation, co-expression networks, tissue-specific programs, 3D genome architecture, and human complex-trait genetics, together with sample distribution across tissues and species.

**Figure S8. Example output page for *CRH* (ENSG00000147571) in brain.** The result page displays four modules: (1) corCor values for human-mouse and human-pig comparisons; (2) WGCNA module information, including module size and gene overlap ratio across species using human as the reference; (3) tissue specificity results, including tau, top tissue, top expression level, and tissue-specificity status; and (4) TAD category details, including cross-species overlap/conservation metrics and genes located within the human TAD region.

**Figure S9.** **Trait and phenotype annotations panel.** The panel summarizes gene-trait and gene-phenotype evidence associated with the queried gene. Summary cards indicate the total number of evidence records, species-level distribution, and evidence scope. Human evidence was derived from direct gene-trait associations in the GWAS Catalog, whereas mouse and pig evidence was based on ortholog-mapped phenotype or trait annotations from MGI and PigBiobank, respectively. The table below summarizes species-specific traits and phenotypes, including the trait or phenotype name, evidence source, best available P value or evidence count, number of supporting records, representative evidence labels, and PubMed or source links when available.
